# Elevated systemic immune-inflammation index is associated with stroke-associated pneumonia in acute ischemic stroke: a retrospective cohort study

**DOI:** 10.3389/fneur.2025.1651656

**Published:** 2025-09-22

**Authors:** Tingting Duan, Ming Yang, Yiming Zhang, Chunyan Zhu, Zichen Rao

**Affiliations:** ^1^Department of Neurology, The Quzhou Affiliated Hospital of Wenzhou Medical University, Quzhou People’s Hospital, Quzhou, Zhejiang, China; ^2^Department of Endocrinology, The Quzhou Affiliated Hospital of Wenzhou Medical University, Quzhou People’s Hospital, Quzhou, Zhejiang, China

**Keywords:** ischemic stroke, stroke-associated pneumonia, systemic immune-inflammation index, inflammatory biomarkers, risk stratification, non-linear modeling

## Abstract

Stroke-associated pneumonia (SAP) is a frequent complication of acute ischemic stroke (AIS) that contributes to poor clinical outcomes. The systemic immune-inflammation index (SII), derived from neutrophil, lymphocyte, and platelet counts, may reflect post-stroke immune imbalance, but its role in predicting SAP remains unclear. In this retrospective study, we analyzed 1,767 AIS patients and evaluated the association between log₂-transformed SII and the occurrence of SAP using multivariable logistic regression, generalized additive models, and two-piecewise regression. SAP developed in 21.3% of patients during hospitalization. Higher SII levels were independently associated with increased SAP risk after adjustment for age, sex, vascular risk factors, comorbidities, baseline National Institutes of Health Stroke Scale (NIHSS) score, and dysphagia assessed by Kubota Water Drinking Test (KWDT). Patients in the highest SII quartile had a significantly greater likelihood of developing SAP compared to those in the lowest quartile (adjusted odds ratio = 2.03, 95% confidence interval: 1.21–3.38, *p* = 0.0069). A non-linear, threshold-dependent relationship was identified, with SAP risk increasing substantially beyond log₂-SII ≈ 8.5. Receiver operating characteristic (ROC) analysis demonstrated moderate predictive performance of SII for SAP (area under the curve (AUC) = 0.726), while C-reactive protein (CRP) showed superior discrimination (AUC = 0.826 *p* < 0.0001). Supplementary sensitivity analyses, including a fully adjusted model without NIHSS and KWDT and an alternative model replacing these with the A2DS2 score (Age, Atrial fibrillation, Dysphagia, Sex, Stroke Severity), showed consistent results, supporting the robustness of our findings. These findings suggest that SII may serve as a cost-effective and accessible biomarker to aid early identification of high-risk AIS patients.

## Introduction

Stroke remains one of the leading causes of mortality and long-term disability worldwide, with acute ischemic stroke (AIS) accounting for approximately 80% of all cases (1). Despite advances in acute stroke management, complications during hospitalization, particularly stroke-associated pneumonia (SAP), continue to pose significant challenges (2). SAP occurs in 10 to 30% of AIS patients and is closely associated with prolonged hospital stays, increased healthcare costs, and worse functional outcomes, including higher mortality rates (3). Early identification of high-risk patients is essential to guide preventative interventions and improve prognosis (4).

Emerging evidence suggests that systemic inflammation plays a pivotal role in the development of SAP by disrupting immune homeostasis and enhancing susceptibility to pulmonary infections following AIS (5). Conventional inflammatory biomarkers such as CRP, white blood cell (WBC) count, and neutrophil-to-lymphocyte ratio (NLR) have been widely used to assess systemic inflammation; however, their predictive accuracy for SAP remains suboptimal (6). The systemic immune-inflammation index (SII) calculated from platelet count, neutrophil count, and lymphocyte count, is a novel composite marker that reflects the balance between pro-inflammatory and immune-regulatory responses (7). Recent studies have demonstrated its prognostic value in various cardiovascular and oncologic conditions, but its predictive utility in SAP remains underexplored (8). In this study, we focused on SII as a comprehensive marker of immune-inflammatory balance, and compared it with CRP, a widely used reference biomarker in stroke research, to assess whether SII provides additional or complementary prognostic value beyond CRP. Moreover, existing studies evaluating inflammatory markers in SAP have primarily focused on linear relationships, potentially overlooking complex non-linear and threshold effects (9). For example, Kuang et al. (10) examined the association between SII and SAP risk in a smaller, mixed cohort of acute stroke patients and reported a linear relationship, without investigating potential non-linear patterns or thresholds. Whether elevated SII levels exhibit a dose–response relationship or specific thresholds beyond which SAP risk dramatically increases has not been fully elucidated. Addressing these knowledge gaps is critical for refining clinical risk stratification and informing targeted preventative strategies (11).

We posited that early elevation of the SII, as a marker of post-stroke immune disequilibrium, would identify AIS patients at independently higher risk of SAP, and that the exposure–response might be non-linear with a clinically relevant threshold. To test this hypothesis, we investigated the association between SII and the development of SAP in patients with AIS by analyzing a large, retrospective cohort. Specifically, we examined the predictive value of log₂-transformed SII, explored potential non-linear and threshold effects through advanced modeling approaches, and compared the diagnostic performance of SII with established inflammatory biomarkers such as CRP.

## Materials and methods

### Study design and participants

This study utilized data from a previously established retrospective cohort investigating the prognosis of ischemic stroke. Patient data collected at The Quzhou Affiliated Hospital of Wenzhou Medical University (Quzhou People’s Hospital), Zhejiang, China, between September 2016 and September 2022 that met the inclusion criteria were included. All eligible patients were enrolled in a single-center retrospective cohort study and were subsequently classified into two groups based on the development of SAP during hospitalization: the SAP group and the non-SAP group. No case–control matching was performed, and group differences were addressed using multivariable models. This study was approved by the Ethics Committee of Quzhou People’s Hospital (Approval Number: 2023-151), which granted a waiver of informed consent due to the retrospective nature of the study and the use of de-identified data. The data were accessed for analysis on April 26, 2025. Prior to analysis, all data were anonymized, and researchers did not have access to any personally identifiable information.

Inclusion criteria were as follows: Patients were included if they met all of the following criteria: (1) Confirmed diagnosis of AIS within 24 h of symptom onset, with admission during the same time window; (2) Brain magnetic resonance imaging (MRI) performed within 48 h of symptom onset using standard sequences (including diffusion-weighted imaging, fluid-attenuated inversion recovery, susceptibility-weighted imaging, T1-weighted, T2-weighted, and magnetic resonance angiography), with radiological confirmation of acute infarction; (3) Blood samples for neutrophil-to-lymphocyte ratio (NLR) and platelet count were obtained within 48 h of symptom onset to assess the acute immune-inflammatory status; (4) Complete clinical and laboratory data available for calculation of the SII.

Exclusion criteria included: (1) Severe systemic comorbidities, including hepatic dysfunction (alanine aminotransferase >10 × or aspartate aminotransferase >3 × the upper limit of normal), renal impairment (serum creatinine >443 μmol/L), active malignancies, or hematologic disorders; (2) Cardiopulmonary insufficiency, including New York Heart Association class III–IV heart failure, left ventricular ejection fraction <40%, chronic obstructive pulmonary disease, or respiratory tract infection at admission; (3) Active infections at admission, including respiratory (e.g., community-acquired pneumonia [CAP]), urinary, or systemic infections; (4) Pregnancy or lactation; (5) Inability to reliably assess stroke severity due to coma, severe aphasia, or other neurologic deficits precluding use of the National Institutes of Health Stroke Scale (NIHSS); (6) Multiple AIS hospitalizations during the study period (only the first admission was included); (7) Missing laboratory data required for calculation of SII (i.e., Platelet count, Neutrophil count or Lymphocyte count); (8) Patients diagnosed with autoimmune diseases.

**Figure 1:**
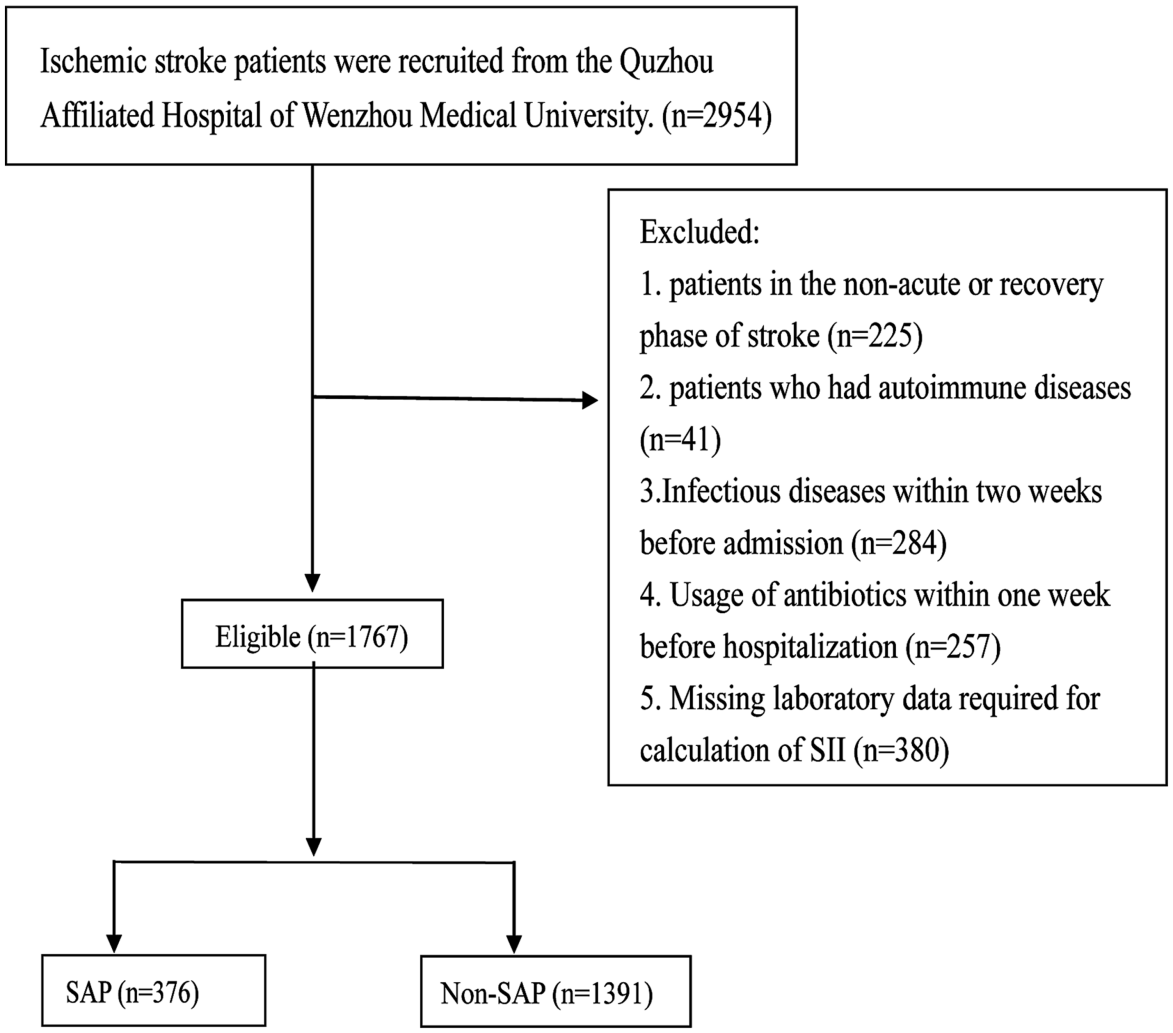
Patient selection flowchart for the study of the systemic immune-inflammation index (SII) and stroke-associated pneumonia (SAP). A total of 2,954 ischemic stroke patients were screened at the Quzhou Affiliated Hospital of Wenzhou Medical University. Patients were excluded if they were in the non-acute or recovery phase of stroke (*n* = 225), had autoimmune diseases (*n* = 41), had infectious diseases within 2 weeks prior to admission (*n* = 284), had received antibiotics within 1 week before hospitalization (*n* = 257), or had missing laboratory data required for SII calculation (*n* = 380). Ultimately, 1,767 eligible patients were included and stratified into the SAP group (*n* = 376) and the non-SAP group (*n* = 1,391). SII, systemic immune-inflammation index; SAP, stroke-associated pneumonia.

The detailed patient selection process is illustrated in Figure 1.

### Baseline data collection

Baseline data were retrieved from medical records and included demographic and clinical characteristics at admission, such as age, sex, smoking status, hypertension, type 2 diabetes, AF and chronic obstructive pulmonary disease (COPD). Stroke severity was assessed using the NIHSS, swallowing difficulty was evaluated using the Kubota Water Drinking Test (KWDT), and consciousness disturbances were assessed using the Glasgow Coma Scale (GCS).

Blood samples were collected by trained nurses on the second morning after admission (6:00 AM) using vacuum tubes, stored at 4 °C, and processed within 2 h by certified laboratory technicians. Laboratory tests included: White blood cell count (WBC), Neutrophil-to-lymphocyte ratio (NLR), Platelet count, Aspartate transaminase (AST), Alanine transaminase (ALT), Glycated hemoglobin (HbA1c), Homocysteine (HCY), Serum creatinine (Scr), Albumin (ALB), Triglycerides (TG), Total cholesterol (TC), High-density lipoprotein (HDL-c), Low-density lipoprotein (LDL-c), The neutrophil-to-lymphocyte ratio (NLR) was calculated as neutrophil count divided by lymphocyte count. All laboratory results were reported using standard international units: WBC and PLT in ×10^9^/L, CRP in mg/L, and NLR as a ratio. Reference ranges (e.g., WBC: 4.0–10.0 × 10^9^/L, PLT: 150–400 × 10^9^/L, CRP: <5 mg/L) were provided for clinical interpretation.

To ensure consistency in exposure measurement, only patients who underwent blood testing within 48 h of symptom onset were included. All NIHSS evaluations were performed by neurologists trained in standardized stroke assessment and blinded to laboratory data. Inter-rater reliability was maintained by duplicate scoring in a subset of cases.

### Definitions

The diagnosis of SAP was determined independently by two attending neurologists following the Pneumonia in Stroke Consensus Group recommendations (12). If necessary, an attending respiratory physician was consulted for confirmation. To ensure incident SAP, patients with pneumonia present at admission (i.e., community-acquired pneumonia, CAP) were excluded, and only pneumonia developing after admission within the acute phase following stroke was classified as SAP.

The diagnostic criteria required at least one of the following:

Fever (>38 °C) without an alternative cause;

Abnormal WBC count (leukopenia <4 × 10^9^/L or leukocytosis >12 × 10^9^/L);

Altered mental status in patients ≥70 years without other causes.

Plus at least two of:

Purulent sputum or a change in sputum character;

Increased respiratory secretions or suction needs;

New or worsening cough, dyspnea, or tachypnea (>25 breaths/min);

Auscultatory findings of rales, crackles, or bronchial breath sounds;

Oxygen desaturation (PaO₂/FiO₂ ≤ 240) or increased oxygen requirement;

Radiologic confirmation was required with two consecutive chest X-rays showing new or progressive infiltrates, consolidation, or cavitation. In patients without prior pulmonary or cardiac disease, a single conclusive chest radiograph was deemed sufficient. Given the study’s focus on ischemic stroke-associated pneumonia, chest CT imaging was utilized instead of chest X-rays, providing superior diagnostic clarity. These criteria were selected for their relevance to the study population and their validated effectiveness in pneumonia diagnosis among stroke patients.

The SII was calculated as:

𝑆𝐼𝐼=Platelet count×Neutrophil count/Lymphocyte count.

The estimated glomerular filtration rate (eGFR) was calculated using the Chronic Kidney Disease Epidemiology Collaboration (CKD-EPI) formula to assess renal function.

### Statistical analysis

All statistical analyses were performed using R Studio (version 4.2.2; R Foundation for Statistical Computing, Vienna, Austria) and EmpowerStats (version 2.0; https://www.empowerstats.net). EmpowerStats served as a user-friendly interface that automatically generates and executes standard R code. To ensure reproducibility, key analyses were also re-run directly in R, and the results were identical. The normality of continuous variables was assessed using the Kolmogorov–Smirnov test. Normally distributed variables were expressed as mean ± standard deviation (SD) and compared with Student’s *t*-test, while non-normally distributed variables were presented as median (IQR) and compared using the Mann–Whitney *U* test. Categorical variables were analyzed using the Chi-square or Fisher’s exact test, as appropriate.

To examine the association between SII and SAP, SII values were log₂-transformed to reduce skewness and enhance interpretability. In addition to treating log₂-transformed SII as a continuous variable, we categorized it into quartiles based on its distribution to reduce the influence of extreme values, detect non-linear or threshold effects without prespecifying a cut-off, and facilitate risk comparison between groups in line with prior literature. We analyzed log₂-SII both as a continuous variable and under non-linear frameworks: the continuous approach enabled comparability with prior studies and provided an overall effect estimate, whereas non-linear analyses (GAM and two-piecewise logistic regression) were used to identify and characterize potential threshold-dependent relationships.

Multicollinearity among covariates was assessed using variance inflation factors (VIF). Logistic regression models were used to calculate odds ratios (ORs) and 95% confidence intervals (CIs). Three models were constructed:

Model 1: Unadjusted;

Model 2: Adjusted for age and sex;

Model 3: Further adjusted for smoking status, hypertension, diabetes mellitus, atrial fibrillation (AF), chronic obstructive pulmonary disease (COPD), systolic and diastolic blood pressure (SBP, DBP), uric acid (UA), white blood cell count (WBC), alanine aminotransferase (ALT), aspartate aminotransferase (AST), glycated hemoglobin (HbA1c), estimated glomerular filtration rate (eGFR), baseline NIHSS score (continuous), and KWDT results.

In supplementary sensitivity analyses, we examined two alternative adjustment strategies: (1) a fully adjusted model excluding NIHSS and KWDT; and (2) a model in which age, sex, AF, NIHSS, and KWDT were replaced by the A2DS2 score, to assess the robustness of the results.

Generalized additive models (GAMs) with penalized splines were used to model the relationship between log₂-SII and SAP, allowing for flexible assessment of potential non-linear effects. To identify a threshold, we fitted two-segment logistic regression models across all possible cut points within the observed log₂-SII range. The cut point with the highest log-likelihood was selected as the threshold, and its 95% confidence interval was estimated using 1,000 bootstrap resamples. The predictive performance of SII alone and CRP alone was evaluated using univariate ROC curve analysis, with comparisons of area under the curve (AUC) performed via the DeLong test. To assess the incremental value of SII beyond established predictors, we constructed multivariable logistic regression models including both SII and CRP, and both SII and the A2DS2 score (treated as a continuous variable), and compared their AUCs with those of CRP alone and the A2DS2 score alone, respectively, using the DeLong test. A two-sided *p*-value < 0.05 was considered statistically significant.

## Results

### Baseline characteristics by SAP status

Among the 1,767 AIS patients, 376 (21.3%) developed SAP (Table 1). Compared with the non-SAP group, SAP patients were older (74.49 ± 11.74 vs. 67.86 ± 12.27 years, *p* < 0.001), and more frequently had atrial fibrillation (35.11% vs. 11.50%) and COPD (15.69% vs. 4.03%). No significant differences were observed in sex distribution or smoking status.

**Table 1:** Baseline characteristics of the study population stratified by SAP and non-SAP groups.

Variables	Overall	Non-SAP	SAP	*P*-value
*N*	1767	1,391	376	
Age (years)	69.27 ± 12.45	67.86 ± 12.27	74.49 ± 11.74	**<0.001**
SBP (mmHg)	152.26 ± 21.46	152.21 ± 21.59	152.46 ± 20.96	0.807
DBP (mmHg)	82.64 ± 12.94	82.88 ± 12.98	81.76 ± 12.78	0.239
Gender, *n* (%)				0.134
Female	716 (40.52%)	551 (39.61%)	165 (43.88%)	
Male	1,051 (59.48%)	840 (60.39%)	211 (56.12%)	
Current smoking, *n* (%)				0.990
No	1,118 (63.27%)	880 (63.26%)	238 (63.30%)	
Yes	649 (36.73%)	511 (36.74%)	138 (36.70%)	
Hypertension, *n* (%)				0.786
No	409 (23.15%)	320 (23.01%)	89 (23.67%)	
Yes	1,358 (76.85%)	1,071 (76.99%)	287 (76.33%)	
Diabetes, *n* (%)				0.749
No	1,139 (64.46%)	894 (64.27%)	245 (65.16%)	
Yes	628 (35.54%)	497 (35.73%)	131 (34.84%)	
Atrial fibrillation, *n* (%)				**<0.001**
No	1,475 (83.47%)	1,231 (88.50%)	244 (64.89%)	
Yes	292 (16.53%)	160 (11.50%)	132 (35.11%)	
COPD, *n* (%)				**<0.001**
No	1,652 (93.49%)	1,335 (95.97%)	317 (84.31%)	
Yes	115 (6.51%)	56 (4.03%)	59 (15.69%)	
Laboratory parameters				
HDL (mmol/L)	1.17 ± 0.32	1.16 ± 0.30	1.20 ± 0.35	**0.029**
LDL (mmol/L)	2.85 ± 1.00	2.87 ± 0.99	2.80 ± 1.04	0.128
FPG (mg/dL)	101.70 (90.00–128.43)	99.90 (89.28–124.74)	108.90 (93.06–138.73)	**<0.001**
HbA1C (%)	6.00 (5.50–7.20)	6.00 (5.50–7.20)	6.10 (5.60–7.30)	0.153
TG (mg/dL)	113.37 (80.16–155.00)	117.80 (84.14–163.85)	93.00 (69.08–127.76)	**<0.001**
TC (mmol/L)	4.28 (3.66–5.01)	4.32 (3.69–5.03)	4.18 (3.57–4.92)	**0.022**
SII index	388.52 (255.09–601.37)	352.41 (242.93–506.37)	624.92 (378.67–1065.05)	**<0.001**
CRP (mg/L)	2.82 (1.15–6.89)	2.00 (1.00–4.25)	13.62 (4.00–37.47)	**<0.001**
AST (U/L)	19.00 (16.00–24.95)	19.00 (15.30–24.00)	21.00 (17.00–29.00)	**<0.001**
ALT (U/L)	17.00 (12.00–25.00)	17.00 (12.00–25.00)	16.00 (12.00–25.00)	0.617
BUN (mmol/L)	5.20 (4.17–6.45)	5.10 (4.11–6.26)	5.70 (4.40–7.30)	**<0.001**
UA (umol/L)	312.00 (251.00–380.00)	313.00 (257.40–382.05)	303.75 (235.45–369.80)	**0.034**
EGFR (ml/min/1.73 m^2^)	99.31 (79.67–118.94)	100.69 (81.77–119.34)	93.86 (71.75–116.24)	**<0.001**
HCY (umol/L)	14.92 (11.71–19.20)	14.60 (11.50–19.00)	16.30 (12.93–20.02)	**<0.001**
WBC (*10^9^/L)	6.97 (5.60–8.68)	6.60 (5.40–8.00)	9.24 (7.44–11.39)	**<0.001**
Clinical characteristics				
KWDT	1.00 (1.00–1.00)	1.00 (1.00–1.00)	1.00 (1.00–4.00)	**<0.001**
A2DS2	3.00 (2.00–4.00)	2.00 (2.00–4.00)	4.00 (3.00–7.00)	**<0.001**
GCS	15.00 (14.00–15.00)	15.00 (15.00–15.00)	14.00 (12.00–15.00)	**<0.001**
mRS	1.00 (0.00–2.00)	1.00 (0.00–2.00)	3.00 (1.00–5.00)	**<0.001**
NHISS	3.00 (1.00–6.00)	2.00 (1.00–5.00)	5.00 (2.00–13.00)	**<0.001**

Values are presented as mean ± SD, median (IQR), or *n* (%), as appropriate. *p*-values were calculated using *t*-test, Mann–Whitney *U* test, or chi-square test as applicable. *p*-values < 0.05 were considered statistically significant. Bold values indicate statistical significance (*p* < 0.05).

CRP, C-reactive protein; SII, systemic immunity-inflammation index; NIHSS, National Institutes of Health Stroke Scale; SBP, systolic blood pressure; DBP, diastolic blood pressure; FPG, fasting plasma glucose; TG, triglycerides; HbA1C, glycated hemoglobin A1c; TC, total cholesterol; HDL, high-density lipoprotein cholesterol; LDL, low-density lipoprotein cholesterol; AST, aspartate aminotransferase; ALT, alanine aminotransferase; UA, uric acid; BUN, blood urea nitrogen; HCY, homocysteine; WBC, white blood cell count; EGFR, estimated glomerular filtration rate; GCS, Glasgow Coma Scale; KWDT, Kubota Water Drinking Test; A2DS2, Age, atrial fibrillation, dysphagia, stroke severity and sex; AF, atrial fibrillation; COPD, chronic obstructive pulmonary disease.

Laboratory findings showed elevated inflammatory markers in the SAP group, including SII (624.92 [378.67–1065.05] vs. 352.41 [242.93–506.37]), CRP, and WBC. Additionally, fasting glucose, BUN, AST, and homocysteine levels were higher, while triglycerides and eGFR were lower (all *p* < 0.05).

SAP patients exhibited more severe neurological impairment, as indicated by higher NIHSS and mRS scores and lower GCS scores (all *p* < 0.001). In addition, they also had higher A2DS2 scores, consistent with their increased risk of poststroke pneumonia (all *p* < 0.001).

### Baseline characteristics by log₂-SII quartiles

Patient characteristics across log₂-SII quartiles are shown in Table 2. Age and diastolic blood pressure did not significantly differ among the groups. The incidence of SAP increased markedly across quartiles, from 11.1% in Q1–Q2 to 17.2% in Q3 and 45.7% in Q4 (*p* < 0.001). Higher SII quartiles were associated with increasing systolic blood pressure (*p* = 0.002) and a higher proportion of female patients (*p* = 0.023).

**Table 2:** Baseline characteristics of patients stratified by quartiles of log_2_-transformed systemic immunity-inflammation index (SII).

SII log_2_ quartile	Q1	Q2	Q3	Q4	*P*-value
*N*	442	441	442	442	
SAP, *n* (%)	49 (11.09%)	49 (11.11%)	76 (17.19%)	202 (45.70%)	**<0.001**
Age (years)	69.64 ± 11.72	68.42 ± 12.79	69.01 ± 12.57	70.01 ± 12.70	0.242
SBP (mmHg)	149.69 ± 22.10	151.43 ± 21.09	152.98 ± 20.95	154.95 ± 21.38	**0.002**
DBP (mmHg)	81.47 ± 12.65	82.59 ± 12.78	82.66 ± 13.70	83.86 ± 12.54	0.056
Gender, *n* (%)					**0.023**
Female	170 (38.46%)	160 (36.28%)	183 (41.40%)	203 (45.93%)	
Male	272 (61.54%)	281 (63.72%)	259 (58.60%)	239 (54.07%)	
Current smoking, *n* (%)					0.148
No	276 (62.44%)	262 (59.41%)	286 (64.71%)	294 (66.52%)	
Yes	166 (37.56%)	179 (40.59%)	156 (35.29%)	148 (33.48%)	
Hypertension, *n* (%)					0.118
No	119 (26.92%)	100 (22.68%)	101 (22.85%)	89 (20.14%)	
Yes	323 (73.08%)	341 (77.32%)	341 (77.15%)	353 (79.86%)	
Diabetes, *n* (%)					0.103
No	268 (60.63%)	294 (66.67%)	278 (62.90%)	299 (67.65%)	
Yes	174 (39.37%)	147 (33.33%)	164 (37.10%)	143 (32.35%)	
Atrial fibrillation, *n* (%)					**<0.001**
No	381 (86.20%)	389 (88.21%)	369 (83.48%)	336 (76.02%)	
Yes	61 (13.80%)	52 (11.79%)	73 (16.52%)	106 (23.98%)	
COPD, *n* (%)					**0.013**
No	415 (93.89%)	417 (94.56%)	421 (95.25%)	399 (90.27%)	
Yes	27 (6.11%)	24 (5.44%)	21 (4.75%)	43 (9.73%)	
Laboratory parameters					
HDL (mmol/L)	1.17 ± 0.31	1.13 ± 0.28	1.15 ± 0.34	1.22 ± 0.32	**<0.001**
LDL (mmol/L)	2.73 ± 0.89	2.80 ± 0.99	2.93 ± 1.03	2.94 ± 1.07	**0.003**
FPG (mg/dL)	99.81 (88.38–123.75)	97.92 (87.84–122.94)	101.70 (90.54–128.07)	108.36 (93.78–137.34)	**<0.001**
HbA1C (%)	6.10 (5.60–7.30)	5.90 (5.50–7.10)	6.10 (5.50–7.19)	6.01 (5.50–7.30)	0.286
TG (mg/dL)	116.03 (79.93–161.86)	123.11 (86.80–176.25)	113.81 (84.36–154.78)	94.33 (73.51–131.97)	**<0.001**
TC (mmol/L)	4.22 (3.61–4.85)	4.21 (3.57–4.94)	4.33 (3.70–5.08)	4.38 (3.72–5.18)	**0.033**
SII index	202.24 (156.56–228.03)	318.99 (286.19–349.82)	474.84 (430.89–523.95)	899.58 (705.21–1210.17)	**<0.001**
CRP (mg/L)	2.00 (1.00–4.00)	2.00 (0.98–4.26)	3.00 (1.28–7.00)	6.14 (2.36–18.65)	**<0.001**
AST (U/L)	20.00 (17.00–25.00)	19.00 (16.00–24.00)	19.00 (15.00–24.00)	20.00 (16.00–26.00)	**0.002**
ALT (U/L)	18.00 (13.00–25.00)	16.00 (12.00–25.00)	17.00 (12.00–24.00)	16.00 (12.00–25.00)	**0.028**
BUN (mmol/L)	5.12 (4.16–6.28)	5.20 (4.30–6.21)	5.10 (4.10–6.38)	5.38 (4.08–6.80)	0.140
UA (umol/L)	317.25 (262.00–381.88)	320.00 (257.50–390.00)	305.10 (250.32–372.40)	309.00 (236.48–375.70)	**0.022**
EGFR (ml/min/1.73 m^2^)	99.55 (80.72–119.79)	97.76 (79.50–115.64)	101.95 (80.52–118.21)	100.47 (78.66–122.12)	0.425
HCY (umol/L)	14.45 (11.53–19.20)	14.20 (11.40–18.90)	15.30 (11.92–19.08)	15.85 (12.50–19.67)	**0.006**
WBC (*10^9^/L)	5.78 (4.80–7.16)	6.38 (5.34–7.66)	7.20 (6.00–8.43)	9.12 (7.50–11.27)	**<0.001**
Clinical characteristics					
KWDT	1.00 (1.00–1.00)	1.00 (1.00–1.00)	1.00 (1.00–1.00)	1.00 (1.00–3.00)	**<0.001**
A2DS2	2.00 (2.00–4.00)	2.00 (2.00–4.00)	2.00 (2.00–4.00)	3.00 (2.00–5.00)	**<0.001**
GCS	15.00 (15.00–15.00)	15.00 (15.00–15.00)	15.00 (14.00–15.00)	15.00 (13.00–15.00)	**<0.001**
mRS	1.00 (0.00–2.00)	1.00 (0.00–2.00)	1.00 (0.00–2.00)	2.00 (1.00–4.00)	**<0.001**
NHISS	2.00 (1.00–4.00)	2.00 (1.00–5.00)	3.00 (1.00–5.00)	4.00 (2.00–9.00)	**<0.001**

Values are presented as mean ± SD, median (IQR), or *n* (%), as appropriate. *P*-values were calculated using *t*-test, Mann–Whitney *U* test, or chi-square test as applicable. *p*-values < 0.05 were considered statistically significant. Bold values indicate statistical significance (*P* < 0.05).

SAP, stroke-associated pneumonia; CRP, C-reactive protein; SII, systemic immunity-inflammation index; NIHSS, National Institutes of Health Stroke Scale; SBP, systolic blood pressure; DBP, diastolic blood pressure; FPG, fasting plasma glucose; TG, triglycerides; HbA1C, glycated hemoglobin A1c; TC, total cholesterol; HDL, high-density lipoprotein cholesterol; LDL, low-density lipoprotein cholesterol; AST, aspartate aminotransferase; ALT, alanine aminotransferase; UA, uric acid; BUN, blood urea nitrogen; HCY, homocysteine; WBC, white blood cell count; EGFR, estimated glomerular filtration rate; GCS, Glasgow Coma Scale; KWDT, Kubota Water Drinking Test; A_2_DS_2_, age, atrial fibrillation, dysphagia, stroke severity and sex; AF, atrial fibrillation; COPD, chronic obstructive pulmonary disease.

The prevalence of atrial fibrillation increased from 13.80% in Q1 to 23.98% in Q4 (*p* < 0.001), and COPD was more frequent in higher quartiles (*p* = 0.013). Laboratory findings showed progressively higher fasting glucose, LDL, CRP, and WBC counts and lower triglycerides across quartiles (all *p* < 0.001).

Neurological scores, including NIHSS, A2DS2, and mRS, increased with higher SII quartiles, while GCS scores decreased (all *p* < 0.001). Dysphagia was more common in the higher quartiles (*p* < 0.001).

### Association between log₂-SII and SAP

Multivariable logistic regression analysis results are shown in Table 3. Compared to Q1, the unadjusted odds ratios (ORs) for SAP were 1.67 (95% CI: 1.13–2.45, *p* = 0.0091) for Q3 and 6.75 (95% CI: 4.75–9.59, *p* < 0.0001) for Q4. After adjustment for age and sex, the associations remained significant (Q3: OR = 1.74, 95% CI: 1.17–2.58, *p* = 0.0059; Q4: OR = 7.48, 95% CI: 5.20–10.75, *p* < 0.0001). In the fully adjusted model, only Q4 remained significantly associated with SAP (OR = 2.03, 95% CI: 1.21–3.38, *p* = 0.0069), while Q2 and Q3 were not statistically significant (both *p* > 0.05).

**Table 3:** Multivariable logistic regression analysis of the association between log_2_-transformed SII quartiles and stroke-associated pneumonia (SAP).

Exposure	Crude model (model 1)		Partially adjusted model (model 2)		Fully adjusted model (model 3)	
	OR (95% CI)	*P*-value	OR (95% CI)	*P*-value	OR (95% CI)	*P*-value
Log₂-SII	2.39 (2.09, 2.73)	<0.0001	2.48 (2.17, 2.84)	<0.0001	1.41 (1.17, 1.69)	0.0002
SII quartile						
Q1	1.0		1.0		1.0	
Q2	1.00 (0.66, 1.53)	0.9905	1.04 (0.68, 1.60)	0.8460	1.02 (0.59, 1.75)	0.9486
Q3	1.67 (1.13, 2.45)	**<0.0001**	1.74 (1.17, 2.58)	**0.0059**	1.21 (0.73, 2.03)	0.4603
Q4	6.75 (4.75, 9.59)	**<0.0001**	7.48 (5.20, 10.75)	**<0.0001**	2.03 (1.21, 3.38)	**0.0069**
*P* for trend	**<0.0001**		**<0.0001**		**0.0035**	

Model 1: Unadjusted.

Model 2: Adjusted for age and sex.

Model 3: Adjusted for age, sex, smoking status, hypertension, diabetes, atrial fibrillation (AF), Chronic obstructive pulmonary disease (COPD), Systolic blood pressure (SBP), Diastolic blood pressure (DBP), uric acid (UA), white blood cell count (WBC), Alanine aminotransferase (ALT), Aspartate aminotransferase (AST), Glycated hemoglobin (HbA1c), and estimated glomerular filtration rate (eGFR), baseline NIHSS score (continuous), and KWDT results.

Values are expressed as odds ratio (OR) with 95% confidence interval (CI). *P*-values < 0.05 were considered statistically significant. Bold values indicate statistical significance (*p* < 0.05). Global *p*-values for quartiles were calculated using trend tests.

When modeled as a continuous variable, log₂-SII was significantly associated with SAP across all models. The unadjusted OR was 2.39 (95% CI: 2.09–2.73, *p* < 0.0001); adjusted ORs were 2.48 (95% CI: 2.17–2.84, *p* < 0.0001) in Model 2 and 1.41 (95% CI: 1.17–1.69, *p* = 0.0002) in Model 3. All variance inflation factors (VIFs) were below 3, indicating no evidence of multicollinearity among the covariates (Supplementary Table 2).

In sensitivity analyses, the association between log₂-SII and SAP remained consistent when using a fully adjusted model without NIHSS and KWDT (Supplementary Table 1A) and when replacing age, sex, AF, NIHSS, and KWDT with the A2DS2 score (Supplementary Table 1B). The effect estimates were comparable to those observed in the main model, supporting the robustness of our findings.

### Subgroup analyses

**Figure 2:**
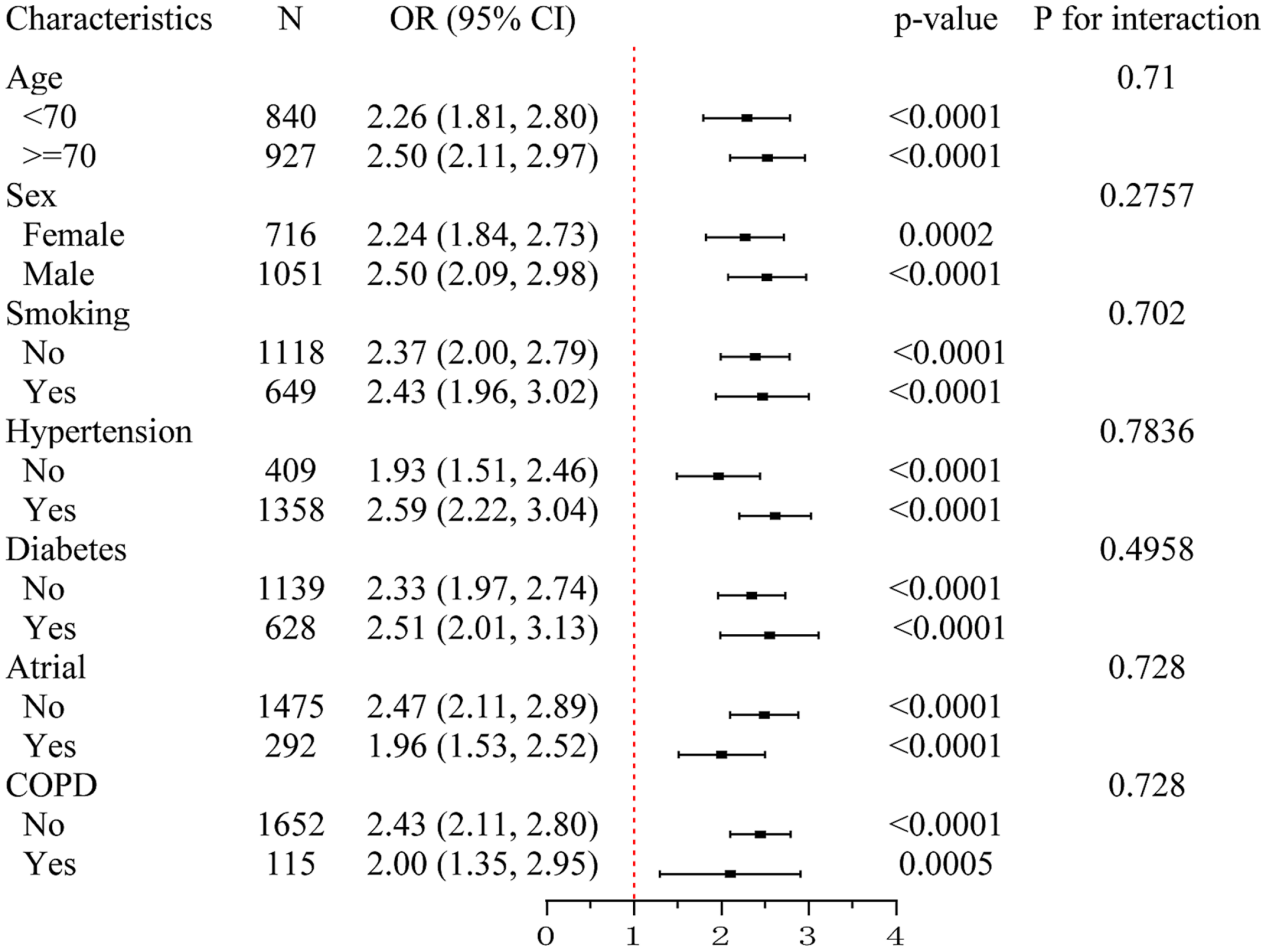
Subgroup analysis of the association between systemic immune-inflammation index (SII) and stroke-associated pneumonia (SAP). This forest plot illustrates odds ratios (ORs) and 95% confidence intervals (CIs) for the association between elevated SII and the risk of SAP across predefined clinical subgroups, including age (<70 vs. ≥70 years), sex, smoking status, hypertension, diabetes, atrial fibrillation, and chronic obstructive pulmonary disease (COPD). Elevated SII was consistently associated with increased SAP risk in all subgroups. No significant interactions were detected (all *P* for interaction > 0.05), suggesting stability of the association across strata. SII, systemic immune-inflammation index; SAP, stroke-associated pneumonia; OR, odds ratio; CI, confidence interval; COPD, chronic obstructive pulmonary disease.

To assess the consistency of the association between elevated SII and SAP, subgroup analyses were conducted across key clinical variables (Figure 2). The association remained significant in both age groups (<70 years: OR = 2.26, 95% CI: 1.81–2.80; ≥70 years: OR = 2.50, 95% CI: 2.11–2.97) and in both sexes (female: OR = 2.24, 95% CI: 1.84–2.73; male: OR = 2.50, 95% CI: 2.09–2.98). Similar results were observed in patients with and without smoking history, hypertension, diabetes, atrial fibrillation, and COPD. All *p* values for interaction were greater than 0.05, indicating that the effect of SII on SAP risk was consistent across clinical subgroups.

### Predictive performance of SII and CRP for stroke-associated pneumonia

**Figure 3:**
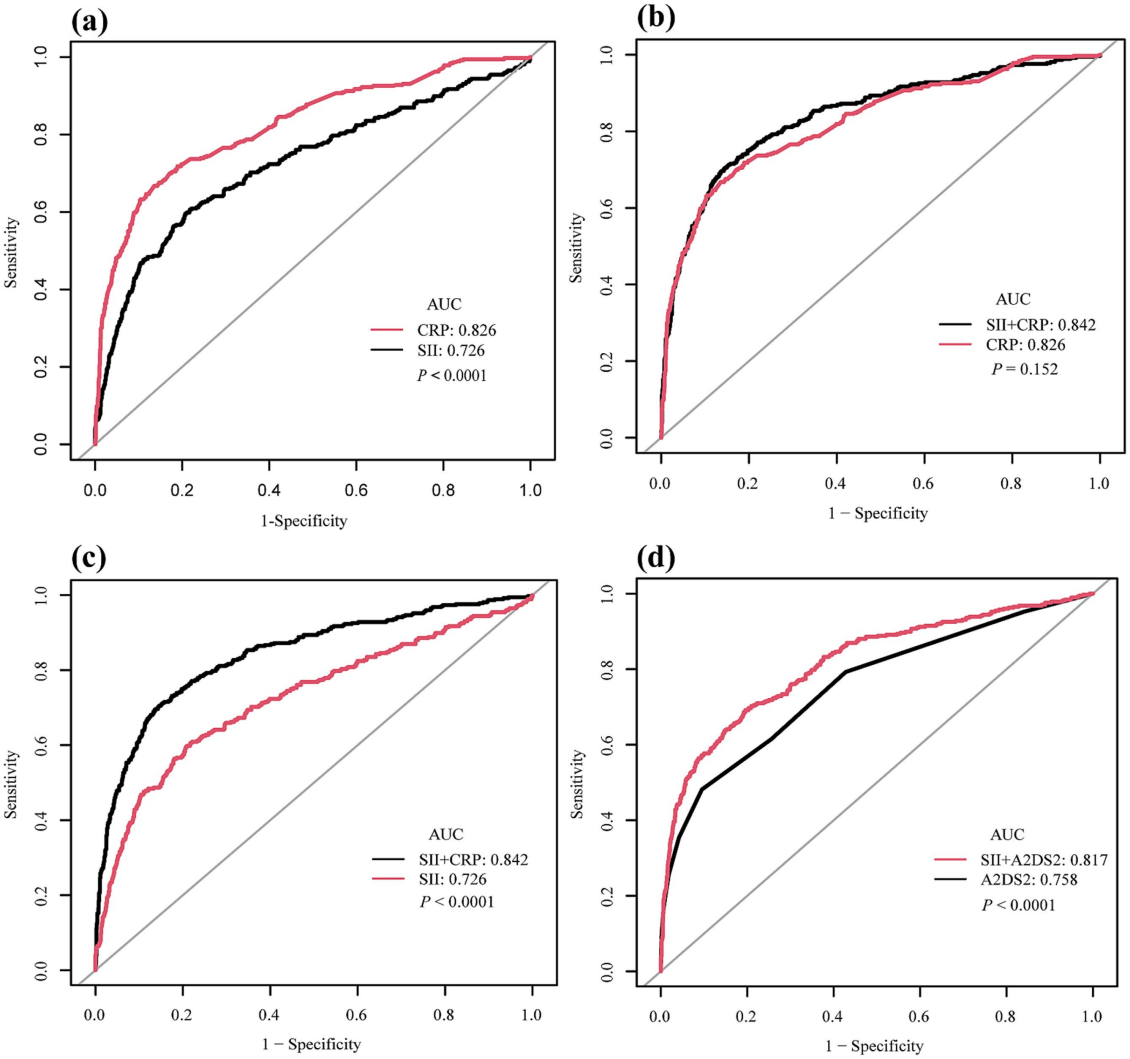
Receiver operating characteristic (ROC) curves for predictive performance of systemic immune-inflammation index (SII), C-reactive protein (CRP), and A2DS2 in relation to stroke-associated pneumonia (SAP). **(a)** Comparison of SII (AUC = 0.726, 95% CI: 0.694–0.758) and CRP (AUC = 0.826, 95% CI: 0.800–0.851) showing CRP had superior discriminatory ability (*p* < 0.0001). **(b)** ROC curves of CRP alone versus the combined model of SII + CRP. The combined model (AUC = 0.842, 95% CI: 0.817–0.866) was not significantly different from CRP alone (*p* = 0.152). **(c)** ROC curves of SII alone versus the combined model of SII + CRP. The addition of SII significantly improved predictive performance compared with SII alone (*p* < 0.0001). **(d)** ROC curves of A2DS2 score alone (AUC = 0.758, 95% CI: 0.729–0.787) versus the combined model of SII + A2DS2 (AUC = 0.817, 95% CI: 0.790–0.843), showing a significant improvement (*p* < 0.0001). ROC, receiver operating characteristic; AUC, area under the curve; SII, systemic immune-inflammation index; CRP, C-reactive protein; A2DS2, Age, Atrial Fibrillation, Dysphagia, Stroke Severity and Sex; SAP, stroke-associated pneumonia.

ROC curve analysis demonstrated that both log₂-transformed SII and CRP had predictive value for SAP in patients with acute ischemic stroke. As shown in Figure 3a, the area under the curve (AUC) was 0.726 (95% CI: 0.694–0.758) for SII and 0.826 (95% CI: 0.800–0.851) for CRP, with a statistically significant difference between the two (*p* < 0.0001) indicating that CRP had a significantly greater discriminatory capacity. At the optimal cutoff point, SII had a sensitivity of 59.6% and a specificity of 79.3%. For CRP, the sensitivity was 66.8% and the specificity was 86.4%. The overall accuracy was 75.1% for SII and 82.2% for CRP.

In an additional analysis, we constructed a multivariable model including both SII and CRP, which yielded an AUC of 0.842 (95% CI: 0.817–0.866). This was not significantly different from that of CRP alone (AUC 0.826, 95% CI: 0.800–0.851, *p* = 0.152) (Figure 3b). Similarly, the AUC of the combined model was significantly higher than that of SII alone (*p* < 0.0001) (Figure 3c). Similarly, when A2DS2 was analyzed as a continuous score, its AUC was 0.758 (95% CI: 0.729–0.787), and adding SII increased the AUC to 0.817 (95% CI: 0.790–0.843) (*p* < 0.0001) (Figure 3d).

Detailed diagnostic metrics for all models (AUC with 95% CI, sensitivity, specificity, and accuracy) are presented in Table 4.

**Table 4:** Comparison of diagnostic performance of SII, CRP, A2DS2, and their combination models for predicting stroke-associated pneumonia.

Model	AUC	95% CI (AUC)	Sensitivity (%)	Specificity (%)	Accuracy (%)
SII	0.726	0.694–0.758	59.6	79.3	75.1
CRP	0.826	0.800–0.851	66.8	86.4	82.2
A2DS2	0.758	0.729–0.787	48.1	90.5	81.5
SII + CRP	0.842	0.817–0.866	70.5	85.5	82.3
SII + A2DS2	0.817	0.790–0.843	69.2	80.5	78.0

Table summarizes the diagnostic performance of log₂-transformed SII, CRP, A2DS2, and their combinations for predicting stroke-associated pneumonia (SAP) in acute ischemic stroke, including the area under the ROC curve (AUC) with 95% confidence interval (CI), sensitivity, specificity, and overall accuracy. Statistical comparisons between AUCs were performed using the DeLong test.

ROC, receiver operating characteristic; AUC, area under the curve; CI, confidence interval; SII, systemic immune-inflammation index; CRP, C-reactive protein; A2DS2, age, atrial fibrillation, dysphagia, sex, stroke severity; SAP, stroke-associated pneumonia.

### Non-linear association and threshold effect of SII on SAP risk

When log₂-SII was treated as a continuous variable in the fully adjusted logistic regression model, a significant overall positive association with SAP was observed (OR = 1.98 per 1-unit increase, 95% CI: 1.62–2.42, *p* < 0.001; Table 5), providing an overall effect estimate for comparability with prior studies.

**Table 5:** Threshold effect and linear association between log₂-SII and stroke-associated pneumonia.

SAP Outcome	Adjusted OR (95% CI) *P*-value
SII (Model I) Linear association (log₂-SII)	1.50 (1.26–1.79) **< 0.0001**
SII (Model II)	
Inflection point	8.48
SII < 8.48	0.80 (0.59, 1.08) 0.1420
SII > =8.48	2.20 (1.72, 2.81) **< 0.0001**
Difference (Segment 2-1)	2.75 (1.77, 4.27) **< 0.0001**
Log likelihood ratio	**<0.001**

Multivariable logistic regression models evaluating the linear and two-piecewise associations between log₂-transformed systemic immune-inflammation index (SII) and the risk of stroke-associated pneumonia (SAP). Model I assumes a linear effect, while Model II incorporates a threshold at the inflection point (*K* = 8.48). Estimates are adjusted for age, sex, smoking status, hypertension, diabetes mellitus, atrial fibrillation (AF), chronic obstructive pulmonary disease (COPD), systolic blood pressure (SBP), diastolic blood pressure (DBP), uric acid (UA), white blood cell count (WBC), alanine aminotransferase (ALT), aspartate aminotransferase (AST), glycated hemoglobin (HbA1c), and estimated glomerular filtration rate (eGFR).

SII, systemic immune-inflammation index; SAP, stroke-associated pneumonia; OR, odds ratio; CI, confidence interval.Bold values indicate statistical significance (*p* < 0.05).

A two-piecewise logistic regression model identified an inflection point at log₂-SII = 8.48. Below this threshold, SII was not significantly associated with SAP (OR = 0.80, 95% CI: 0.59–1.08, *p* = 0.1420), whereas above the threshold, a significant association was observed (OR = 2.20, 95% CI: 1.72–2.81, *p* < 0.0001). The difference in effect between segments was statistically significant (OR = 2.75, 95% CI: 1.77–4.27, *p* < 0.0001), and the log-likelihood ratio test supported the superiority of the threshold model over the linear model (*p* < 0.001) (Table 5). The threshold was determined by the cut point with the highest log-likelihood (95% CI obtained via 1,000 bootstrap resamples; see Methods).

**Figure 4:**
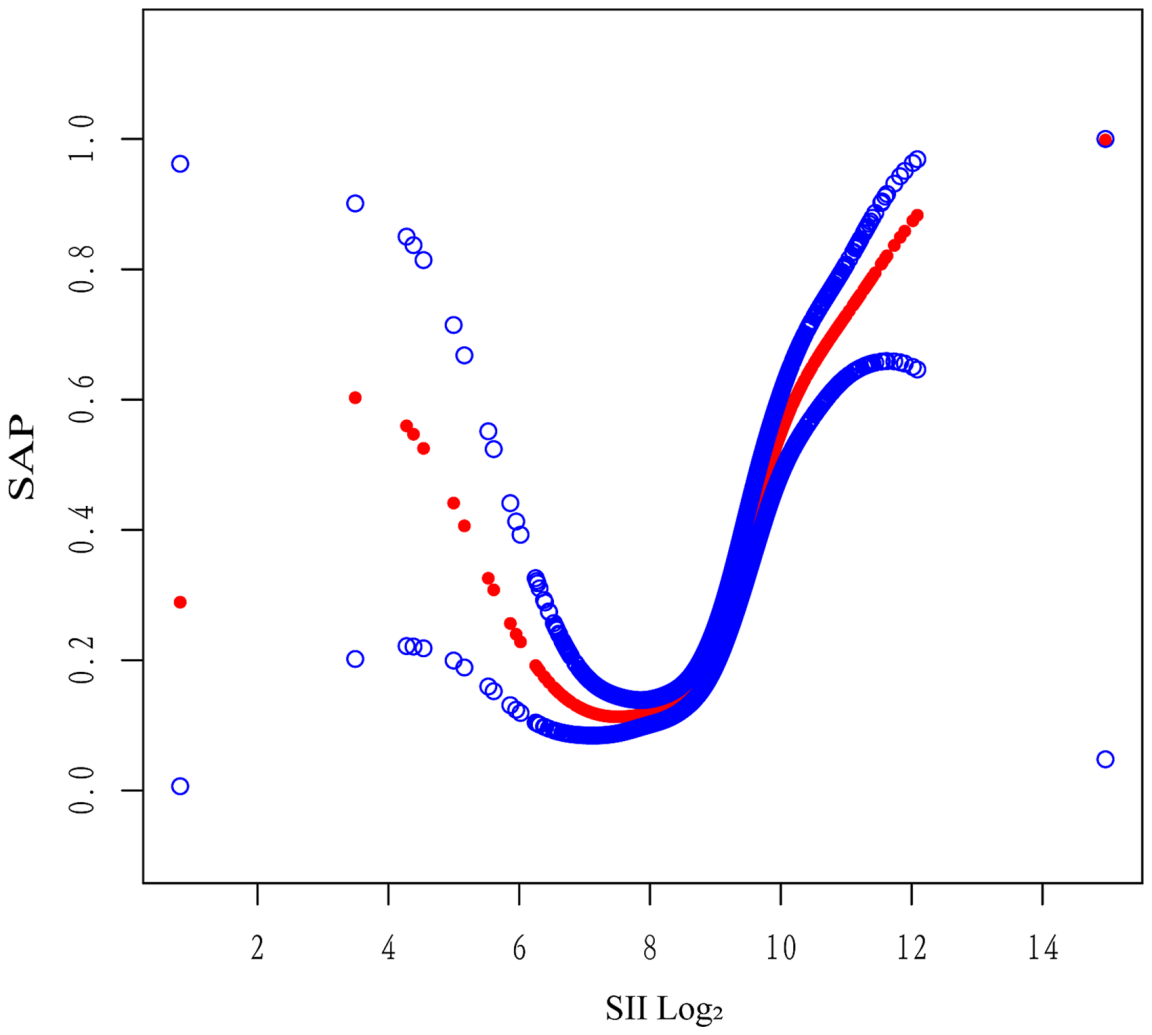
Generalized additive model illustrating the non-linear relationship between log₂-SII and SAP risk. The generalized additive model (GAM) depicts a non-linear association between log₂-transformed systemic immune-inflammation index (SII) and the probability of stroke-associated pneumonia (SAP). The SAP risk remained relatively stable at lower SII levels and increased sharply beyond log₂-SII ≈ 8.5, indicating a threshold-dependent relationship. The model was adjusted for relevant covariates described in the main analysis. SII, systemic immune-inflammation index; SAP, stroke-associated pneumonia; GAM, generalized additive model.

Generalized additive model (GAM) analysis further supported a non-linear association between log₂-SII and SAP risk, revealing a flat trend at lower SII levels and a sharp increase beyond log₂-SII ≈ 8.5, consistent with the identified inflection point (Figure 4).

## Discussion

In this retrospective cohort of 1,767 patients with AIS, elevated SII levels were independently associated with an increased risk of SAP. Patients in the highest SII quartile had a significantly greater risk of SAP (adjusted OR = 2.31, 95% CI: 1.44–3.70; *p* = 0.0005) compared to those in the lowest quartile. Furthermore, GAM and two-piecewise logistic regression identified a non-linear, threshold-dependent relationship between log₂-SII and SAP risk, indicating that the association becomes more pronounced beyond a specific threshold. To facilitate comparability with prior studies, we also reported the effect estimate from a model treating log₂-SII as a continuous predictor; this should be interpreted as an average effect across the exposure range, whereas the non-linear analyses (GAM and two-segment logistic regression) better characterize the threshold-dependent pattern observed (Table 5 and Figure 4).

The association between elevated SII and SAP risk may reflect stroke-induced immune dysfunction (13). AIS activates neuroendocrine responses such as the hypothalamic–pituitary–adrenal (HPA) axis and sympathetic nervous system, leading to increased levels of glucocorticoids and catecholamines (14, 15). These hormonal shifts promote lymphocyte apoptosis and suppression of adaptive immunity, characteristic of stroke-induced immunodepression syndrome (SIDS) (16, 17). By integrating neutrophil, platelet, and lymphocyte counts, SII captures this imbalance-reflecting both overactivation of innate immunity (neutrophilia and thrombocytosis) and suppression of adaptive responses (lymphopenia) (18–20).

Beyond stroke, SII has demonstrated prognostic relevance in various clinical conditions (21, 22). In cardiovascular disease, elevated SII levels have been independently associated with poor outcomes in coronary artery disease (23, 24), as well as with all-cause and cardiovascular mortality in large-scale cohorts (25–27). In oncology, SII has emerged as a reliable biomarker of systemic inflammation and prognosis across multiple malignancies, including hepatocellular, colorectal, and gynecologic cancers (22, 28–31). Furthermore, in sepsis, SII has shown predictive value for both disease severity and mortality, reinforcing its utility as a broad indicator of immune dysregulation (32, 33).

SII may also act as a surrogate marker for deeper immunopathological processes involved in the development of SAP (34, 35). Neutrophilia promotes the release of reactive oxygen species and proteases that damage the alveolar-capillary barrier (36, 37). Concurrent lymphopenia compromises adaptive immunity, while elevated platelet counts enhance inflammation through cytokine release (38, 39). This triad generates a pro-inflammatory yet immunosuppressed state, predisposing patients to pulmonary infections (40, 41).

Additional evidence from post-stroke immunology studies indicates that peripheral lymphocyte counts, particularly T cells, B cells, and NK cells, decline rapidly within hours to days after AIS onset due to apoptosis, redistribution to lymphoid organs, and functional exhaustion (42, 43). This lymphopenia, a hallmark of stroke-induced immunodepression syndrome (SIDS), compromises adaptive immunity and reduces pathogen clearance capacity (42, 44), thereby increasing susceptibility to infections such as SAP. In parallel, neutrophil and platelet activation further amplifies systemic inflammation and damages the alveolar-capillary barrier (45) (with NETs broadly detected in AIS thrombi (46)), creating a “double-hit” effect of immune suppression and inflammatory injury. These dynamic changes in immune cell populations directly influence SII values, as a rising SII often reflects both increased innate immune activation (neutrophilia, thrombocytosis) and marked adaptive immune suppression (lymphopenia) (42, 43), providing a plausible mechanistic link between elevated SII and heightened SAP risk in AIS patients.

Unlike CRP, SII reflects cellular immune dynamics by integrating neutrophils, platelets, and lymphocytes (47). This composite measure captures both innate activation and adaptive suppression (7). The threshold-dependent pattern observed in our study suggests that SAP risk rises notably only when this imbalance exceeds a critical level. CRP, as an acute-phase reactant produced by the liver in response to infection or tissue injury, often rises rapidly and directly with the onset of infection—this may explain its higher discriminatory performance for SAP in our cohort (e.g., Liu et al. found that CRP levels within 12 h of stroke onset were independently associated with poor outcomes) (48, 49). In contrast, SII reflects the underlying immune-inflammatory balance and may be more informative for early risk stratification, before overt infection occurs. Although other inflammatory markers such as WBC and NLR have been studied—NLR may help identify high-risk SAP patients (50)—these markers typically show modest and heterogeneous discrimination (6). Therefore, we prioritized SII as an integrative index and used CRP as a reference biomarker; WBC was adjusted for in models, and SII remained independently associated with SAP (Table 3; VIFs < 3).

Previous studies, such as Kuang et al. (10), have reported a linear association between SII and SAP risk in a mixed acute stroke population, but did not explore potential non-linear patterns or threshold effects and did not restrict the analysis to a well-defined cohort of AIS patients. By applying GAM and two-piecewise logistic regression, our study identified a distinct inflection point, revealing that the relationship between SII and SAP is not uniform across its range. Furthermore, our work extends prior findings by evaluating the added predictive value of SII beyond established clinical severity scores (NIHSS and A2DS2), thereby providing a more nuanced understanding of how immune imbalance contributes to SAP development and underscoring the value of advanced modeling in biomarker-based risk stratification.

These findings not only enhance our understanding of SII’s prognostic relevance but also support its potential integration into clinical risk models. Compared with biomarkers like CRP, SII is more accessible, cost-effective, and readily obtained from routine blood tests. Combining SII with validated scores such as A2DS2 could improve early SAP detection, particularly in resource-limited settings (51, 52). Future studies should explore dynamic SII monitoring and assess its predictive value across different stroke subtypes and care settings (53).

Several limitations should be acknowledged. First, the retrospective and single-center design may limit generalizability. Second, inflammatory markers were measured only once within 48 h of admission, precluding evaluation of temporal changes. Third, although our main models adjusted for baseline stroke severity (NIHSS) and dysphagia (KWDT), residual confounding by unmeasured aspects of severity (e.g., level of consciousness, aspiration risk, use of nasogastric/jejunal tubes, prolonged bed rest, and infarct location) cannot be fully excluded despite multivariable adjustment. In addition, we did not evaluate other inflammatory or immune biomarkers (e.g., cytokines or measures of immune cell function), which may provide complementary information beyond SII and CRP and should be explored in future work. Finally, long-term outcomes, such as SAP recurrence or post-discharge mortality, were not assessed. Prospective, multicenter studies are warranted to validate our findings and further refine the clinical utility of SII.

## Conclusion

In this retrospective cohort of AIS patients, elevated SII levels were independently and non-linearly associated with SAP risk, exhibiting a clear threshold effect. Although CRP demonstrated superior discriminative ability, SII showed complementary prognostic value, particularly when combined with CRP or A2DS2, while remaining easily obtainable from standard blood counts. Integrating SII into existing clinical risk models may enhance early identification of high-risk individuals. Prospective, multicenter investigations are warranted to validate these findings and to explore whether dynamic SII monitoring can improve both short- and long-term outcomes in AIS populations.

## Data Availability

The original contributions presented in the study are included in the article/Supplementary material, further inquiries can be directed to the corresponding author.

## Glossary

AIS: acute ischemic stroke
SAP: stroke-associated pneumonia
SII: systemic immune-inflammation index
CRP: C-reactive protein
GAM: generalized additive model
ROC: receiver operating characteristic
AUC: area under the curve
NIHSS: National Institutes of Health Stroke Scale
GCS: Glasgow Coma Scale
KWDT: Kubota Water Drinking Test
A2DS2: Age, Atrial fibrillation, Dysphagia, Sex, Stroke severity
NLR: neutrophil-to-lymphocyte ratio
PLT: platelet count
WBC: white blood cell count
ALT: alanine aminotransferase
AST: aspartate aminotransferase
HbA1c: glycated hemoglobin
HCY: homocysteine
Scr: serum creatinine
ALB: albumin
TG: triglycerides
TC: total cholesterol
HDL-c: high-density lipoprotein cholesterol
LDL-c: low-density lipoprotein cholesterol
AF: atrial fibrillation
COPD: chronic obstructive pulmonary disease
SBP: systolic blood pressure
DBP: diastolic blood pressure
eGFR: estimated glomerular filtration rate
UA: uric acid
MRI: magnetic resonance imaging
CT: computed tomography
SD: standard deviation
OR: odds ratio
CI: confidence interval
