# Supplementary material for: Elevated systemic immune-inflammation index is associated with stroke-associated pneumonia in acute ischemic stroke: a retrospective cohort study
Source: Front Neurol. 2025 Sep 22;16:1651656. doi: 10.3389/fneur.2025.1651656 (PMC12497611; doi:10.3389/fneur.2025.1651656)
Supplement: Supplementary file 1 [file Table_1.docx]

**Supplementary Table 1a. Sensitivity analysis: Multivariable logistic regression for the association between log₂-transformed SII quartiles and stroke-associated pneumonia (SAP) — fully adjusted model without NIHSS and KWDT**

| Exposure | Crude Model (Model 1) | | Partially Adjusted Model (Model 2) | | Fully Adjusted Model (Model 3) | |
| --- | --- | --- | --- | --- | --- | --- |
|  | OR (95% CI) | *P*-value | OR (95% CI) | *P*-value | OR (95% CI) | *P*-value |
| Log₂-SII | 2.39 (2.09, 2.73) | **<0.0001** | 2.48 (2.17, 2.84) | **<0.0001** | 1.50 (1.26, 1.79) | **<0.0001** |
| SII quartile |  |  |  |  |  |  |
| Q1 | 1.0 |  | 1.0 |  | 1.0 |  |
| Q2 | 1.00 (0.66, 1.53) | 0.9905 | 1.04 (0.68, 1.60) | 0.8460 | 0.97 (0.58, 1.60) | 0.8914 |
| Q3 | 1.67 (1.13, 2.45) | **<0.0001** | 1.74 (1.17, 2.58) | 0.0059 | 1.21 (0.75, 1.95) | 0.4255 |
| Q4 | 6.75 (4.75, 9.59) | **<0.0001** | 7.48 (5.20, 10.75) | **<0.0001** | 2.31 (1.44, 3.70) | **0.0005** |
| P for trend | **<0.0001** | | **<0.0001** | | **0.0002** | |

Model 1: Unadjusted;

Model 2: Adjusted for age and sex;

Model 3: Adjusted for age, sex, smoking status, hypertension, diabetes, atrial fibrillation (AF), Chronic obstructive pulmonary disease (COPD), Systolic blood pressure (SBP), Diastolic blood pressure (DBP), uric acid (UA), white blood cell count (WBC), Alanine aminotransferase (ALT), Aspartate aminotransferase (AST), Glycated hemoglobin (HbA1c), and estimated glomerular filtration rate (eGFR).

Values are expressed as odds ratio (OR) with 95% confidence interval (CI). *P*-values < 0.05 were considered statistically significant. Bold values indicate statistical significance (p < 0.05).

**Supplementary Table 1b. Sensitivity analysis: Multivariable logistic regression for the association between log₂-transformed SII quartiles and stroke-associated pneumonia (SAP) — adjusted model with A2DS2 instead of age, sex, AF, NIHSS, and KWDT**

| Exposure | Crude Model (Model 1) | | Partially Adjusted Model (Model 2) | | Fully Adjusted Model (Model 3) | |
| --- | --- | --- | --- | --- | --- | --- |
|  | OR (95% CI) | *P*-value | OR (95% CI) | *P*-value | OR (95% CI) | *P*-value |
| Log₂-SII | 2.39 (2.09, 2.73) | **<0.0001** | 2.48 (2.17, 2.84) | **<0.0001** | 1.35 (1.13, 1.62) | **0.0011** |
| SII quartile |  |  |  |  |  |  |
| Q1 | 1.0 |  | 1.0 |  | 1.0 |  |
| Q2 | 1.00 (0.66, 1.53) | 0.9905 | 1.04 (0.68, 1.60) | 0.8460 | 0.99 (0.59, 1.69) | 0.9847 |
| Q3 | 1.67 (1.13, 2.45) | **<0.0001** | 1.74 (1.17, 2.58) | **0.0059** | 1.27 (0.77, 2.09) | 0.3413 |
| Q4 | 6.75 (4.75, 9.59) | **<0.0001** | 7.48 (5.20, 10.75) | **<0.0001** | 1.92 (1.17, 3.14) | **0.0094** |
| P for trend | **<0.0001** | | **<0.0001** | | **0.0039** | |

Model 1: Unadjusted;

Model 2: Adjusted for age and sex;

Model 3: Adjusted for smoking status, hypertension, diabetes, Chronic obstructive pulmonary disease (COPD), Systolic blood pressure (SBP), Diastolic blood pressure (DBP), uric acid (UA), white blood cell count (WBC), Alanine aminotransferase (ALT), Aspartate aminotransferase (AST), Glycated hemoglobin (HbA1c), and estimated glomerular filtration rate (eGFR) and A2DS2 score.

Values are expressed as odds ratio (OR) with 95% confidence interval (CI). *P*-values < 0.05 were considered statistically significant. Bold values indicate statistical significance (p < 0.05).
