# Supplementary material for: Elevated systemic immune-inflammation index is associated with stroke-associated pneumonia in acute ischemic stroke: a retrospective cohort study
Source: Front Neurol. 2025 Sep 22;16:1651656. doi: 10.3389/fneur.2025.1651656 (PMC12497611; doi:10.3389/fneur.2025.1651656)
Supplement: Supplementary file 2 [file Table_2.docx]

**Supplementary Table 2. Variance inflation factors (VIFs) for covariates included in the multivariable logistic regression model**

| **Variable** | **VIF** |
| --- | --- |
| Age | 1.4 |
| Gender | 1.8 |
| Current smoking | 1.8 |
| Hypertension | 1.1 |
| Diabetes | 1.9 |
| Atrial fibrillation | 1.2 |
| COPD | 1.1 |
| HbA1c | 1.9 |
| UA | 1.3 |
| eGFR | 1.5 |
| WBC | 1.4 |
| HDL | 1.1 |
| LDL | 1.1 |
| AST | 2.9 |
| ALT | 2.9 |
| HCY | 1.1 |
| NIHSS score | 1.7 |
| KWDT | 1.8 |

Variance inflation factors (VIFs) were calculated for all covariates included in the fully adjusted logistic regression model. All VIF values were <3, indicating no evidence of multicollinearity. **Abbreviations:** COPD, chronic obstructive pulmonary disease; HbA1c, glycated hemoglobin; UA, uric acid; eGFR, estimated glomerular filtration rate; WBC, white blood cell count; HDL: High-density lipoprotein cholesterol; LDL: Low-density lipoprotein cholesterol; AST, aspartate aminotransferase; ALT, alanine aminotransferase; HCY: Homocysteine; NIHSS, National Institutes of Health Stroke Scale; KWDT, Kubota water swallowing test.
